# Early Gender Differences in Pain and Functional Recovery Following Thoracolumbar Spinal Arthrodesis

**DOI:** 10.3390/jcm10163654

**Published:** 2021-08-18

**Authors:** Matthew T. Gulbrandsen, Nina Lara, James A. Beauchamp, Andrew Chung, Michael Chang, Dennis Crandall

**Affiliations:** 1 Department of Orthopedic Surgery, Loma Linda University, Loma Linda, CA 92354, USA; 2 Department of Orthopedic Surgery, Mayo Clinic, Phoenix, AZ 85054, USA; Nina.J.Lara@gmail.com (N.L.); msc@sonoranspine.com (M.C.); dennis@sonoranspine.com (D.C.); 3 Department of Biomedical Engineering, Northwestern University, Evanston, IL 60208, USA; james.beauchamp@northwestern.edu; 4 Sonoran Spine Center, Tempe, AZ 85281, USA; andrewchung84@gmail.com

**Keywords:** gender differences, spine arthrodesis, spinal fusion, spine, deformity

## Abstract

Background: To analyze gender differences regarding the recovery experience (pain, function, complications) after spinal arthrodesis surgery. Methods: Pre-operative and post-operative gender-based differences in patient-reported outcomes for open posterior spinal arthrodesis at 6 weeks, 3 months, 6 months, and 1 year were studied, including age, comorbidities, body mass index (BMI), diagnosis, number of vertebrae fused, type of surgery, primary vs. revision surgery, and complications. Statistical analysis included the use of Student’s *t*-test, Chi square, linear regression, Mann–Whitney U test, and Spearman’s rho. Results: Primary or revision posterior arthrodesis was performed on 1931 consecutive adults (1219 females, 712 males) for deformity and degenerative pathologies. At surgery, females were older than males (61.7 years vs. 59.7 years, *p* < 0.01), had slightly more comorbidities (1.75 vs. 1.5, *p* < 0.01), and were more likely to undergo deformity correction (38% vs. 22%, *p* < 0.01). Females described more pre-op pain (female VAS = 6.54 vs. male VAS = 6.41, *p* < 0.01) and lower pre-op function (female ODI = 49.73 vs. male ODI = 46.52, *p* < 0.01). By 3 months post-op, there was no significant gender difference in VAS or ODI scores. Similar pain and function scores between males and females continued through 6 months and 12 months. Conclusion: Although females have more pain and dysfunction before undergoing spinal surgery, the differences in these values do not reach the Minimum Clinically Important Difference (MCID). Post-operatively, there is no difference in pain and function scores among males and females at 3, 6, and 12 months.

## 1. Introduction

Historically, common stereotypes exist regarding the differences in how males and females perceive pain. Females have been reported to describe higher levels of pain when presented with equal amounts of thermal stimuli compared to males [1]. Females have also shown a lower threshold for thermal pain and lower pain tolerance than males [2]. Tonelli et al. reported female joint arthroplasty patients experienced more pain and dysfunction than males, even in the setting of less severe osteoarthritis [3].

However, in the setting of low back pain, much is unknown regarding gender perceived differences in pain and functional outcomes. Chenot et al. found that females had a lower pain threshold and lower functional capacity than males with chronic low back pain [4]. On the other hand, females with chronic low back pain treated with spinal fusion have been shown to experience similar pain and functional outcomes when compared to males [5]. For patients undergoing laminectomy alone or with fusion, similar ultimate clinical outcomes have been reported without gender differences [6].

Specifically, gender differences in pain perception and function after spinal fusion surgery have not been studied in the setting of lumbar degenerative disease or thoracolumbar deformity. Consequently, gender-based outcome differences remain unclear in patients undergoing spinal surgery for these conditions. The purpose of this study is therefore to analyze how a patient’s gender impacts self-reported pain and functional recovery after spinal arthrodesis surgery for thoracolumbar deformity and lumbar degenerative disease.

## 2. Materials and Methods

### 2.1. Patient Sample

This was a retrospective cohort study utilizing patient data from a single center’s prospectively collected surgical database that received IRB exemption. Only adult patients (>18 years old) undergoing open posterior instrumented arthrodesis were included in this study. Included were both primary and revision surgeries of any length, with or without interbody fusions, for lumbar degenerative conditions and thoracolumbar deformity. Patients without a minimum 1 year of clinical and radiographic follow-up data were excluded. Trauma, tumor, and infection cases were additional grounds for exclusion. All surgeries were performed by 5 fellowship trained spine surgeons.

A similar strategy for post-operative pain management and limited narcotic use was used throughout this study, with an effort to have all patients off opiate analgesics by 3 months post-op. Post-operative bracing was optional and provided at the request of individual patients. Post-operative physical therapy was typically instituted at 8–12 weeks post-op, and continued for 4 weeks. All patients were placed on a home exercise program after formal physical therapy was completed.

All patient demographic information and baseline characteristics including comorbidities, smoking status, body mass index (BMI), and indication for surgery were noted. Surgical factors were additionally collected.

### 2.2. Outcome Measures

Clinical outcome measurements included the Visual Analogue Scale (VAS) and the Oswestry Disability Index (ODI). These scores were collected pre-operatively, at 6 weeks, 3 months, 6 months, and 1 year after surgery. Radiographic data were additionally collected at similar time points. Radiographic evidence of fusion included no implant–bone interface lucency, apparent bridging bone either posterolaterally or through the interspace, and no motion on flexion-extension radiographs at the 1 year post-operative follow-up. All peri-operative complications were noted.

### 2.3. Statistical Analysis

Mann–Whitney U test, was employed to determine potential gender differences in ODI and VAS scores. Student’s *t*-test was used to compare gender differences in age at the time of surgery. Spearman’s rho analysis was used to determine the strength of association between either VAS or ODI scores and a patient’s gender, BMI, and age. A Chi-Square test was used to determine potential gender differences in the presence of complications, type of diagnosis (degenerative vs. deformity), and number of comorbidities. Linear regression models were used to estimate and compare the differential effects of a patient’s gender, diagnosis (degenerative vs. deformity), age, number of comorbidities, BMI, levels of fusion, revision status, and presence of complications on ODI and VAS scores over time. Statistical significance was set at *p* < 0.01. All statistical analyses were conducted with IBM SPSS Statistics for Windows (IBM Corp., Armonk, NY, USA).

## 3. Results

### 3.1. Patient Characteristics

A total of 1931 consecutive patients (female: 1219, male: 712) met inclusion criteria. Mean follow-up was 84 months; range, 12–192 months) (Table 1). Males had a slightly higher BMI than females (29.7 vs. 28.7; *p* < 0.01) and were more likely to require surgery for degenerative disease compared to other diagnoses (78% vs. 62%; *p* < 0.01) (Table 2). Females tended to be older than males at the time of surgery (61.7 years ± 12.8 vs. 59.7 years ± 14.1; *p* < 0.01). In general, females had a greater number of comorbidities compared to males (1.75 vs. 1.5; *p* < 0.01, Table 3). Comorbidities included in this study were autoimmune disorders, gastrointestinal disorders, depression, fibromyalgia, and thyroid disease. There was no difference in smoking status between groups (*p* > 0.01).

A total of 1045 patients (54.1%) underwent primary fusion (Table 1). There were no statistical differences in levels fused between the two groups. On average, males with deformity disease underwent 8.2 level fusions and females underwent 8.64 level fusions. For degenerative disease, males underwent 2.09 level fusions and females underwent 2.13 level fusions.

### 3.2. Pain and Function

Females reported slightly higher pain scores pre-operatively (6.54 vs. 6.14; *p* < 0.01). At 6 weeks post-op, females continued to describe marginally more pain than males (VAS 4.36 vs. 3.99; *p* < 0.01). By 3 months, there was no gender-based difference in pain scores (female VAS: 3.73 vs. male VAS: 3.76; *p* > 0.01). Furthermore, there was no significant gender difference in pain scores at 6 months (*p* > 0.01), or 1 year post-operatively (*p* > 0.01). Both male and female patients demonstrated significant clinical improvement in pain scores by 1 year follow-up (Table 4).

Females reported lower pre-op dysfunction scores (ODI scores, Table 4) when compared to males (F = 49.73 vs. M = 46.52; *p* < 0.01). Functional improvements in both genders were significant at 1 year (*p* < 0.01). At one year, there was no gender difference in ODI scores noted (*p* > 0.01). Females experienced a slightly greater mean overall improvement in ODI by 1 year (20 points in females vs. 16.6 points for males; *p* < 0.01).

### 3.3. Gender-Based Complication Rates

Comparing post-operative complications in our study group, there were no gender differences in pseudarthrosis rates, re-operation rates, or other complications (Table 5). Death is listed as a complication for any patient who died within 2 years of surgery.

### 3.4. Predictors of Pain and Function

Predictors of pain and function were estimated through a linear regression model of 1 year post-operative VAS scores (Table 7) and ODI scores (Table 6), respectively. Separate models were fit for each gender and prediction estimates were based on the parameter coefficients (β), with significant coefficients (*p* < 0.05) interpreted as the estimated change in VAS or ODI for a unit change in the respective factor. As can be seen in Table 6, number of comorbidities, type of diagnosis, presence of complications, and BMI were found to significantly contribute to female 1 year post-operative ODI scores, while number of comorbidities, level of fusion, type of diagnosis, presence of complications, and BMI were found to significantly contribute to male 1 year post-operative ODI scores. Likewise, as shown in Table 7, number of comorbidities, presence of complications, BMI, and age were found to significantly contribute to female 1 year post-operative VAS scores, while number of comorbidities, level of fusion, type of diagnosis, presence of complications, and age were found to significantly contribute to male 1 year post-operative VAS scores.

## 4. Discussion

Numerous studies suggest certain patient characteristics and comorbidities affect outcomes after spinal fusion [7,8,9,10,11,12]. The few risk factors that have been shown to consistently result in worse outcomes include BMI, age, cardiovascular disease, smoking, and receiving worker’s compensation or disability benefits [7,8,9,10]. However, the effect of patient gender on outcome after spinal arthrodesis has not been solidified.

In 2002, Gehrchen et al. conducted a retrospective review including 112 patients with degenerative disc disease (DDD) and spondylolisthesis that showed female gender to be an independent risk factor for a nonoptimal outcome after lumbar fusion [12]. In 2009, Ekman conducted a randomized control trial that included 164 patients treated with spinal fusion for spondylolisthesis that suggested females had worse PROs post-operatively [10]. In 1984, when analyzing the outcomes after treatment for cervical disc disease, Eriksen et al. found that females have more pain and dysfunction post-operatively after fusion surgery [13].

However, the results of our study align more closely to those of Triebel et al. and Pochon et al. [5,6]. Triebel et al., in a study that included 4780 Swedish patients with lumbar degenerative disc disease and chronic low back pain, found that Swedish women reported similar pain and function outcomes to men after lumbar spinal fusion [5]. Additionally, a 2016 study by Pochon et al. that included 1518 patients found that females who underwent decompression alone or decompression with fusion ± instrumentation did not experience a difference in outcomes when compared to men [6].

Our study shows that while females reported slightly more pain and worse function than males at the time of surgery, by 3 months and beyond, no further gender differences in post-operative pain or function existed. Our findings support the ultimate conclusions of gender outcome equality by Triebel et al. and Pochon et al. However, our study further expands their findings to the realm of deformity surgery [5,6].

An important aspect to acknowledge when reviewing the results of our study is the MCID for VAS back pain and ODI score. Previous studies have suggested that the MCID for VAS and ODI are 2.1 and 14.9, respectively [14,15]. Both MCIDs are significantly higher than the difference found in at any time point in our study. Therefore, the slightly increased pain (F = 6.54 vs. M = 6.14; *p* < 0.01) and disability (F = 49.73 vs. M= 46.52; *p* < 0.01) that females present with prior to undergoing spinal arthrodesis is not clinically relevant.

Similar results have been echoed in the total joint arthroplasty literature. For instance, Holtzman and Katz showed that females have more pain and dysfunction prior to undergoing total joint arthroplasty. However, they found that females do not recover as well post-operatively compared to their male counterparts [16,17]. Another finding of our study was that females were slightly older than males when they underwent spinal arthrodesis (61.7 years ± 12.8 vs. 59.7 years ± 14.1; *p* < 0.01). As far as we are aware, why females wait longer and endure more pain before undergoing spine or total joint surgery has not been well studied. Possible explanations for this phenomenon include that (1) females are more reluctant to choose surgical intervention [18], (2) females spend more time gathering information about risks and benefits [19], and (3) females are more likely than males to be prescribed anti-depressants or referred to mental health before being offered surgical intervention [2,20]. Another possible explanation for delayed spinal arthrodesis in females is that many gender comparative studies performed prior to 2010 showed inferior outcomes in females after spine surgery which may differentially impact the decision making from the surgeon’s standpoint [9,10,12,13].

Given the higher comorbidity burden of females, it is surprising that they ultimately achieved similar outcomes to males. There are several potential explanations. Physical therapy use has been associated with improved outcomes after lumbar fusion [21], and current literature shows that females are much more likely to utilize physical therapy [22,23]. Females are also more likely to follow-up with their physician after lumbar surgery [24]. Interestingly, a study analyzing patient compliance after total knee arthroplasty showed that females are more like to be compliant when compared to males [25]. Female patients’ propensity to attend physical therapy and comply with a physician’s recommendation may explain their increased margin of post-operative improvement compared to males. Once again, it is important to note that although this margin of improvement is statistically significant, it does not reach the MCID and, therefore, is unlikely to be clinically significant.

There are several limitations to this study. First, this study was retrospective in nature. All patients underwent open posterior spinal arthrodesis and results may differ for other approaches or decompression without fusion. Furthermore, patient-reported pain and functional scores are individually subjective. Additionally, although our analyses accounted for many variables, possible confounding variables that we were unable to account for include patient expectations, the operating surgeon, physical therapy effort by the patient, psychosocial factors, living environment, and psychological background. Additionally, this study focused on general VAS scores for pain and did not distinguish between back pain and leg pain. Additionally, the findings here are limited to a single center’s experience and may not be broadly applicable.

## 5. Conclusions

Although females have more pain and dysfunction before undergoing spinal arthrodesis for thoracolumbar deformity and lumbar degenerative disease, the differences in these values do not reach the Minimum Clinically Important Difference (MCID). Post-operatively, there is no difference in pain and function scores among males and females at 3, 6, and 12 months.

## Figures and Tables

**Table 1:** Patient characteristics separated by gender.

Characteristics	Male *n* = 712	Female *n* = 1219	*p* Value
Age (years)	59 ± 14.07	61 ± 12.83	<0.01
Pre-op BMI (kg/m^2^)	29.7	28.7	<0.01
Degenerative ^#^	562 (78.9%)	765 (62.8%)	<0.01
Deformity	150 (21.1%)	454 (37.2%)	<0.01
Smoker	146 (20.5%)	154 (12.6%)	NS
Revision Surgery	334 (46.9%)	552 (45.3%)	NS
Prior Laminectomy	128 (18.0%)	167 (13.6%)	NS
Prior Fusion	206 (28.9%)	385 (31.6%)	NS
Deformity Average Levels Fused	8.20	8.64	NS
Degenerative Average Levels Fused	2.09	2.13	NS

^#^ Degenerative pathology includes degenerative and spondylolisthesis diagnoses. NS = not statistically significant.

**Table 2:** Comparison of patient diagnosis by gender.

Diagnosis	Male *n* = 712	Female *n* = 1219	*p* Value
Degenerative	287 (40.3%)	324 (26.6%)	<0.01
Spondylolisthesis	275 (38.6%)	441 (36.2%)	NS
Adult Idiopathic Scoliosis	20 (2.80%)	135 (11.1%)	<0.01
Degenerative Scoliosis	48 (6.74%)	162 (13.3%)	<0.01
Scheuermann’s Kyphosis	18 (2.52%)	5 (0.41%)	<0.01
Neuromuscular Scoliosis	2 (0.28%)	7 (0.57%)	NS
Other Kyphosis	41 (5.76%)	77 (6.32%)	NS
Kyphoscoliosis	21 (2.95%)	68 (5.58%)	<0.01

**Table 3:** Patient comorbidities separated by gender.

Comorbidities	Male *n* = 712	Female *n* = 1219	*p* Value
Number of Comorbidities	1.50 ± 1.33	1.75 ± 1.42	<0.01
Rheumatoid Arthritis, Systemic Lupus Erythematosus	34 (4.78%)	82 (6.73%)	NS
Bowel/Bladder Dysfunction	33 (4.63%)	93 (7.63%)	NS
Cancer	80 (11.2%)	127 (10.4%)	NS
Stroke, Transient Ischemic Attack	18 (2.53%)	37 (3.04%)	NS
Pulmonary	101 (14.2%)	223 (18.3%)	NS
Vascular Disease	63 (8.85%)	145 (11.9%)	NS

**Table 4:** Visual Analog Scale (VAS) for pain pre-operatively and at 6 weeks, 3 months, 6 months, and 1 year post-operatively, separated by gender. Oswestry Disability Index (ODI) score pre-operatively and post-operatively at 1 year, separated by gender.

	Male *n* = 712	Female *n* = 1219	*p* Value
VAS pre-op (mean)	6.14 (std = 2.16)	6.54 (std = 2.12)	<0.01
VAS 6 weeks (mean)	3.99 (std = 2.32)	4.36 (std = 2.29)	<0.01
VAS 3 months (mean)	3.76 (std = 2.38)	3.73 (std = 2.32)	NS
VAS 6 months (mean)	3.58 (std = 2.57)	3.61 (std = 2.45)	NS
VAS 1 year (mean)	3.50 (std = 2.61)	3.47 (std = 2.61)	NS
Change in VAS from pre-op to 1 year	−2.65 (std = 2.78)	−3.06 (std = 2.82)	<0.01
Pre-operative ODI (mean)	46.52 (std = 16.19)	49.73 (std = 16.44)	<0.01
1 year post-operative ODI (mean)	29.9 (std = 21.6)	29.79 (std = 20.89)	NS
Change in ODI from pre-op to 1 year post-op	−16.63 (std = 18.48)	−20.01 (std = 19.29)	<0.01

**Table 5:** Complications separated by gender.

Complications	Male (*n* = 712)	Female (*n* = 1219)	*p* Value
Nonunion	16 (2.25%)	53 (4.35%)	NS
Adjacent Level Fracture	9 (1.26%)	23 (1.89%)	NS
Implant Loosening	3 (0.42%)	10 (0.82%)	NS
Implant Failure	5 (0.70%)	9 (0.74%)	NS
Neuro Deficit	7 (0.98%)	10 (0.82%)	NS
Death	14 (1.97%)	21 (1.72%)	NS
Deep Venous Thrombosis	3 (0.42%)	3 (0.25%)	NS
Pulmonary Embolus	1 (0.14%)	4 (0.33%)	NS
Deep Infection	22 (3.09%)	27 (2.21%)	NS
Iliac Screw Removal	6 (0.84%)	28 (2.30%)	NS
Revision Laminectomy	25 (3.51%)	46 (3.77%)	NS
Revision Fusion	42 (5.90%)	97 (7.96%)	NS

**Table 6:** Linear regression model of post-op ODI at 1 year for male and female.

	Male 1 Year Post-Operative ODI			Female 1 Year Post-Operative ODI		
	β	Standard Error	*p*-Value	β	Standard Error	*p*-Value
Constant	11.562	5.866	0.049	15.711	4.109	<0.0005
Comorbidities	1.283	0.650	0.049	2.056	0.441	<0.0005
Level of Fusion (Single: 0, Multi: 1)	6.453	1.886	0.001	0.709	1.583	0.654
Diagnosis (Degen.: 0, Deformity: 1)	−4.282	2.094	0.041	−2.876	1.367	0.036
Complication (None: 0, Complications: 1)	3.440	1.638	0.036	4.474	1.188	<0.0005
Body Mass Index	0.483	0.151	0.001	0.481	0.095	<0.0005
Age at Operation	−0.056	0.061	0.364	−0.082	0.048	0.088

**Table 7:** Linear regression model of post-op VAS at 1 year for male and female.

	Male 1 Year Post-Operative Visual Analog Score			Female 1 Year Post-Operative Visual Analog Score		
	β	Standard Error	*p*-Value	β	Standard Error	*p*-Value
Constant	3.057	0.720	<0.0005	2.992	0.526	<0.0005
Comorbidities	0.213	0.079	0.007	0.229	0.056	<0.0005
Level of Fusion (Single: 0, Multi: 1)	0.475	0.231	0.040	0.179	0.203	0.377
Diagnosis (Degen.: 0, Deformity: 1)	−0.732	0.255	0.004	−0.341	0.175	0.051
Complication (None: 0, Complications: 1)	0.535	0.200	0.008	0.359	0.152	0.018
Body Mass Index	0.020	0.019	0.270	0.032	0.012	0.009
Age at Operation	−0.016	0.007	0.035	−0.017	0.006	0.007

## Data Availability

Raw data are from a private clinic database, not publicly available.
